# AGING SEXUAL STEREOTYPES AND SEXUAL EXPRESSION IN MID- AND LATER LIFE: EXAMINING THE STEREOTYPE MATCHING EFFECT

**DOI:** 10.1093/geroni/igz038.3251

**Published:** 2019-11-08

**Authors:** Maggie Syme, Tracy Cohn

**Affiliations:** 1 Kansas State University, Manhattan, Kansas, United States; 2 Radford University, Radford, Virginia, United States

## Abstract

Ageist sexual stereotypes are culturally embedded and may prohibit midlife and older adults from achieving sexual wellness when internalized over the life course (i.e., stereotype embodiment), which was examined in the current study. A cross sectional, convenience sample of 972 adults aged 50 and older was recruited online via a crowdsourcing platform. Participants completed an online survey assessing aging sexual stigma and their participation in a spectrum of sexual and intimate behaviors. Two hierarchical linear regressions examined study hypotheses predicting a) sexual and b) intimate behaviors among middle age and older adults, while accounting for several known covariates (e.g., education, relationship status, health). Results suggest that older age (β = -.24, p < .001), being a woman (β = -.29, p < .001), and higher levels of aging sexual stigma (β = -.30, p < 0.001) were associated with less sexual activity (F(19, 945) = 32.51, p < .001, R2 = 0.40). For intimate behaviors, older age (β = -0.14, p < .05) and higher levels of aging sexual stigma (β = -0.24, p < .001) were significantly associated with lower levels of intimate activity (F(19, 945) = 39.80, p < .001, R2 = 0.45). Contrary to expectations, neither gender nor age cohort moderated the effect of aging sexual stigma. Ageist sexual stereotypes appear to affect individual sexual health and wellness via internalized beliefs. Future studies should focus on the potential malleability of aging sexual stigma beliefs, and at what point(s) in the life course they are modifiable.

